# Single-incision laparoscopic ileocecal resection in a 10-year-old child with appendiceal neuroendocrine tumor

**DOI:** 10.1186/s12957-019-1745-y

**Published:** 2019-11-26

**Authors:** Ayana Goto, Nobuhisa Matsuhashi, Takao Takahashi, Yuta Sato, Shinya Hirata, Toshiyuki Tanahashi, Satoshi Matsui, Hisasi Imai, Yoshihiro Tanaka, Kazuya Yamaguchi, Kazuhiro Yoshida

**Affiliations:** Department of Surgical Oncology, Gifu University School of Medicine, 1-1 Yanagido, Gifu city, 501-1194 Japan

**Keywords:** Appendiceal neuroendocrine tumor, Pediatric, Lymph node metastasis

## Abstract

**Background:**

In Japan, the majority of gastrointestinal tract neuroendocrine tumors (NETs) have been reported to originate from the rectum, and appendiceal NETs are relatively rare. Preoperative diagnosis is very difficult and it is diagnosed after appendectomy. Pediatric appendiceal NET is a disease with a good prognosis. However, in rare cases, lymph node metastasis could occur and additional resection is required.

**Case presentation:**

A 10-year-old boy complained of right lower quadrant abdominal pain and underwent an appendectomy under a diagnosis of acute appendicitis in previous hospital. The final diagnosis was appendiceal NET, so he was referred to our department for additional resection. The tumor was found in the base of the appendix and invasively reached the subserosal layer with obvious vascular invasion. His Ki-67 index was 1 to 2%, so we classified it as appendiceal NET G1 according to the WHO 2015 classification. We considered the possibility of a tumor remnant or lymph node metastasis, so we performed single-incision laparoscopy with D3 lymph node dissection. The pathological diagnosis revealed no tumor remnant but metastasis to one lymph node. He was discharged on the 9th postoperative day. There has been no recurrence at 3 years and 7 months after surgery.

**Conclusion:**

When the tumor size is 10–20 mm, the frequency of lymph node metastasis in some reports is variable, and there is no consensus yet on the indications for additional resection. However, there are definitely a certain number of cases with lymph node metastasis that require additional resection. In the present patient, long-term survival can be obtained by additional resection. At present, factors such as the presence of vascular or lymph node invasion and the malignancy grade and tumor’s location must be considered on a case-by-case basis. Although the incidence rate of appendiceal NET is rare, the diagnosis can be made only during postoperative pathological examination; thus, reliable histopathological examination is required.

## Background

In Japan, the majority of gastrointestinal tract neuroendocrine tumors (NETs) have been reported to originate from the rectum. NETs occurring primarily in the appendix are relatively rare, accounting for just 8–11% of all cases [1]. Pediatric appendiceal NET is associated with a favorable prognosis, and patients requiring additional resection due to lymph node metastasis are extremely rare. We experienced a case of lymph node metastasis from appendiceal NET in a child who underwent additional resection following an appendectomy. We report this case along with a brief review of the literature.

## Case presentation

**Fig. 1:**
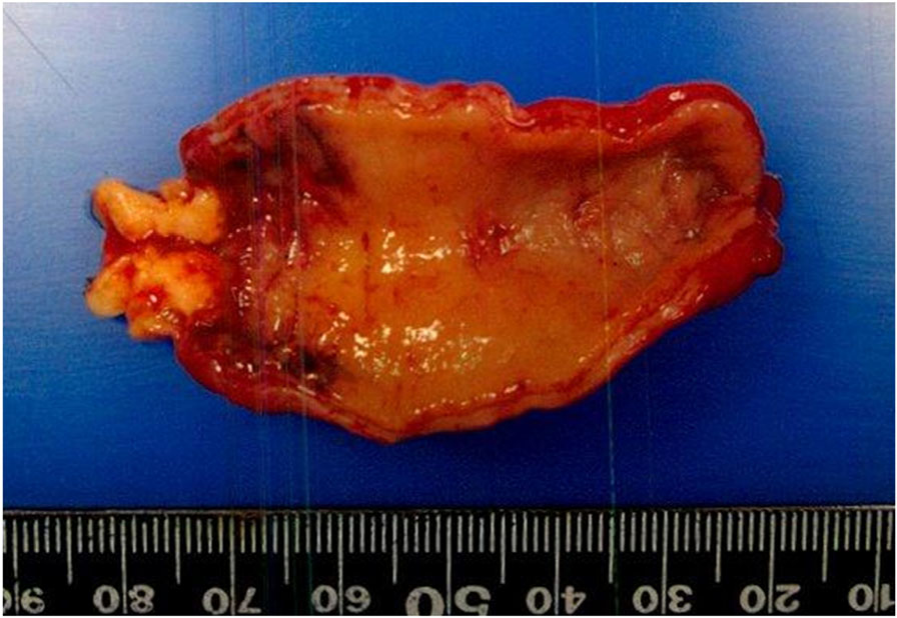
A solid tumor 16 × 15 mm in circumference was detected at the base of the appendix. The peripheral mucosa was partially detached and necrosis was detected

**Fig. 2:**
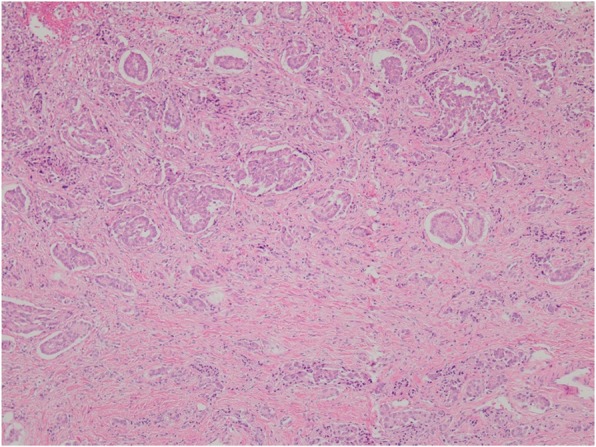
The tumor with island-like, ribbon-shaped and rosette-like alveolar structures was grown

**Fig. 3:**
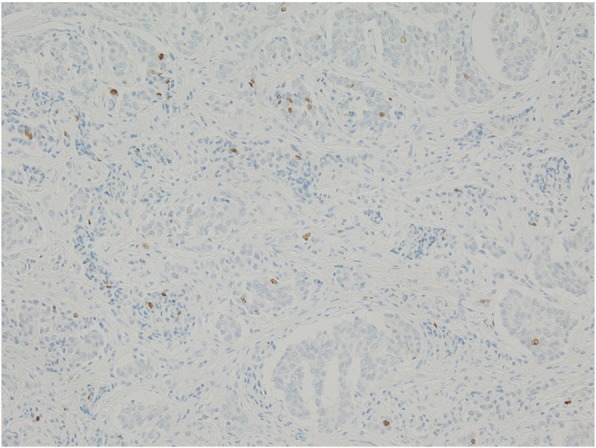
The Ki-67 index was 1–2%

A 10-year-old boy complained of right lower quadrant abdominal pain and underwent an appendectomy under the diagnosis of acute appendicitis at the previous hospital. The pathological diagnosis was appendiceal NET and he was referred to our department for additional resection. In the macroscopically excised specimen at the appendectomy, a solid tumor 16 × 15 mm in circumference was detected at the base of the appendix. The peripheral mucosa was partially detached, and necrosis was detected (Fig. 1). Pathological findings indicated a peripheral tumor at the entrance of the appendix, with the tumor located near the stump. The tumor with island-like, ribbon-shaped, and rosette-like alveolar structures had grown invasively and reached the subserosal layer (Fig. 2). Vascular invasion was evident with a finding of ly2, v1. Immunohistochemical staining was positive for synaptophysin, chromogranin-A, and CD56. The Ki-67 index was 1–2% (Fig. 3). The tumor was classified as an appendiceal NET G1 according to the 2015 World Health Organization (WHO) classification. Preoperative enhanced computed tomography (CT) revealed no lymph node metastasis and small tumor size. However, the possibility of a remnant tumor or lymph node metastasis was considered because the tumor was located near the stump and had invaded the vascular areas. Therefore, a single-incision laparoscopic ileocecal resection with D3 lymph node dissection was performed. A 3-cm skin incision was made in the navel, and surgery was started through a single hole. A 10-cm margin was taken on both the oral and the anal side from the tumor. Since this was a pediatric case, a hand-sewn anastomosis with Albert-Lembert suture was performed rather than a mechanical anastomosis.

The postoperative pathological diagnosis revealed no residual tumor cells in the cecum; however, lymph node metastasis was detected. The postoperative course was uneventful, and he was discharged on the 9th postoperative day. No recurrence has been observed at 3 years and 7 months postoperatively.

## Discussion and conclusion

NET is a relatively rare disease, with 3–5 new cases per 100,000 people diagnosed each year. Obendorfer named this disease small bowel carcinoid in 1907 [2]. Since then, NET has been called a carcinoid tumor and is considered to be benign. However, with increasing clinicopathological studies of NET, the diversity of its malignancy was recognized, and NET was no longer considered a carcinoid, as stated in the 2000 revision of the WHO histopathological classification. Furthermore, in the 2010 revision, a histopathological classification based on tumor grades using the Ki-67 index and number of nuclear fissions that reflect the growth kinetics of tumor cells was established. Since then, gastrointestinal NETs have been classified as grade 1 (G1), grade 2 (G2), and neuroendocrine carcinoma (NEC) [3]. Carcinoid is equivalent to NET G1 according to the 2010 WHO classification. Half of the primary gastrointestinal NETs occur in the rectum, 8–11% occur in the appendix [1], and 0.02–1.5% of NETs have been found in the resected appendix [4]. Appendiceal NET commonly occurs at 42.7 years of age, which is slightly earlier than that of other gastrointestinal NETs because it originates from neuroendocrine cells. Neuroendocrine cells are densely distributed in infancy, and the peak decline in their density occurs at approximately 30 years of age [5]. Approximately 70–90% of NETs are located at the tip of the appendix and its occurrence in the root of the appendix is rare [6]. Carcinoid syndrome rarely occurs in appendiceal NET. Because they present no specific symptoms, most patients are diagnosed after the appearance of symptoms, such as acute appendicitis, or on the basis of pathological findings following an appendectomy. Preoperative diagnosis is extremely difficult.

Basically, if an appendiceal NET is diagnosed, all patients are treated by surgery, and R0 resection is recommended. It is necessary to determine the operative method on case by case because some patients show lymph node metastasis. Tumor diameter has been considered the most reliable factor for lymph node metastasis. It is reported that metastasis does not occur when the diameter is < 10 mm, and in such cases, appendectomy only is sufficient [7]. However, when the tumor diameter exceeds 20 mm, many patients are at high risk for lymph node metastasis. According to the Japanese guidelines, right hemicolectomy or ileocecal resection with D3 lymph node dissection is recommended in these patients [3]. The operative method is controversial for a tumor diameter of 10–20 mm. Although the frequency of lymph node metastasis in this group is usually considered rare, several reports have shown the possibility of lymph node metastasis in this group. Mullen et al. reported that lymph node metastasis was observed in 16 (47%) of their 34 cases [8]. In the Japanese guideline, curative surgery with lymph node dissection is considered possible for lymph node metastasis when NETs measuring 10–20 mm invade the appendix wall, mesoappendix, appendix base, or lymph duct. The tissue type and high grade of Ki-67 index are also indication for curative surgery. Takada et al. reported that they performed additional resection for appendiceal NET with a maximum diameter of 13 mm and lymph duct invasion, and it was positive for lymph node metastasis [9]. In addition, perineural and vascular invasion have been reported to be a risk of lymph node metastasis [10].

In the present patient, considering the possibility of lymph node metastasis, we selected additional resection because the tumor was located at the base of the appendix and showed vascular and lymph duct invasion although the tumor was 16 mm in diameter and lymph node swelling was not evident on preoperative CT.

In a search for pediatric carcinoid/NETs in the database of the Japan Medical Abstracts Society, 15 case reports including our case were found [11]. Four patients underwent additional resection conforming to the current Japanese guidelines, and two patients including our patient showed lymph node metastasis. Moreover, three pediatric patients underwent additional resection after appendectomy in other countries. Among them, lymph node metastasis was observed in one patient with a tumor diameter of 21 mm [12].

The prognosis of pediatric appendiceal carcinoid is good because the appendectomy is performed in childhood in the course of acute appendicitis, and the detected tumor is small in diameter. However, there are some patients with lymph node metastasis of NET G1 same with our case. Therefore, some factors such as the presence of vascular or lymph duct invasion, tumor’s location, and tumor grade must be considered when treating patients with a 10–20-mm tumor diameter. When focusing on the choice of performing an ileocecal resection or right hemicolectomy, it is necessary to examine various factors, such as the grade of malignancy and preoperative CT findings, of each case.

In Japan, laparoscopic surgery for colon cancer is widely accepted, and reduced port surgery has recently become widespread. Takemasa et al. reported that if a skilled surgeon performs single-port operation, the surgical results are equivalent to multiport operation, and postoperative pain is significantly less in single group [13]. In addition, since this patient was a child, we selected single-port operation with consideration of cosmetic side.

As described above, although the incidence of pediatric appendiceal NET is very rare, a certain number of cases require additional resection because of lymph node metastasis.

The tumor diameter in our patient was relatively small at 16 mm, and preoperative CT showed no obvious lymphadenopathy. Therefore, according to some reports, one might think that this patient required no additional resection. However, by performing additional resection, we detected lymph node metastasis that could not be found on preoperative examination, and long-term survival was obtained. Therefore, in daily practice, it is important to perform the curative surgery in consideration of these risk factors in addition to the tumor diameter on case by case.

## Data Availability

Not applicable.

## Abbreviations

CT: Computed tomography
NET: Neuroendocrine tumors
WHO: World Health Organization
